# Intestinal Pioneer Colonizers as Drivers of Ileal Microbial Composition and Diversity of Broiler Chickens

**DOI:** 10.3389/fmicb.2019.02858

**Published:** 2020-01-09

**Authors:** Denise R. Rodrigues, Emily Winson, Kim M. Wilson, Whitney N. Briggs, Audrey F. Duff, Kaylin M. Chasser, Lisa R. Bielke

**Affiliations:** Department of Animal Sciences, The Ohio State University, Columbus, OH, United States

**Keywords:** commensal bacteria, diversity, *Enterobacteriaceae*, *Enterococcus*, immune system, probiotic *in ovo*, segmented filamentous bacteria

## Abstract

Given that recent advances in metagenomics have highlighted the importance of intestinal microbes for poultry health, there has been a corresponding search for early manipulation strategies of intestinal microbiota in order to advance immune system development and optimize functional properties of growth. In this study, we used the *in ovo* technique as an experimental model to address how early bacterial intestinal colonization could affect the development and establishment of the mature ileal microbiota. Inoculations containing one of the following: 0.2 mL of 0.9% sterile saline (S), approximately 10^2^ cells of *Citrobacter freundii* (CF), *Citrobacter* species (C2) or lactic acid bacteria mixture (L) were administered via *in ovo* into the amnion. Results showed that *Enterobacteriaceae* abundance was negatively correlated with aging, although its high population at day of hatch affected the microbiota composition, delaying mature microbiota establishment. L treatment increased colonization of butyrate-producing bacteria by 3 and 10 days, and segmented filamentous bacteria in the lower ileum by 10 days. On the other hand, L-probiotic decreased the population of *Enterococcaceae*. In addition, L and C2 microbial communities were less diverse at 10 than 3 days of age in the upper ileum. Importantly, these findings provide a valuable resource for a potential study model for interactions between microbial colonization and associated immune responses. In conclusion, our analysis demonstrates that intestinal pioneer colonizers play a critical role in driving the course of microbial community composition and diversity over time, in which early life exposure to L-based probiotic supported selection alongside greater colonization of symbiotic populations in the ileum of young broilers.

## Introduction

Resident gastrointestinal (GIT) microbiota, through its interactive metabolic dynamics and immune-inflammatory pathways, may influence both health status and disease susceptibility of the host (Lozupone et al., 2012; Antonissen et al., 2016). Since the importance of intestinal microbes for poultry health has been recognized (Danzeisen et al., 2013; Teague et al., 2017; Johnson et al., 2018), there has been a corresponding search for early manipulation strategies of GIT microbiota in order to advance immune system development and optimize growth performance in poultry. Assembly of the poultry intestinal microbiome starts at hatch with predominant colonization of Firmicutes and Proteobacteria (Ballou et al., 2016; Donaldson et al., 2017). Although the mechanisms by how *Enterobacteriaceae* affect early microbiota are not yet clear, species from this family are typically GIT pioneer colonizers (Lu et al., 2003; Ballou et al., 2016; Wilson et al., 2019). The highly variable intestinal bacterial colonization in chicks may have inadvertently resulted from the hatchery microbiota, which represents a critical environmental source for newly hatched chicks and will serve as the basis from which the intestinal microbial communities will settle at a later age (Stanley et al., 2013; Ballou et al., 2016; Rubio, 2018; Kubasova et al., 2019).

Dynamics of the microbial establishment may also be affected by dietary supplementation of lactic acid bacteria (LAB). Several studies have shown that the addition of probiotics in the feed represents an effective method for improving growth performance, enhancing humoral immunity and accelerating the maturation of intestinal microbiota (Liao et al., 2012; Berni Canani et al., 2016; Donaldson et al., 2017; Rubio, 2018). Nevertheless, many of the reports regarding broilers have focused on long-term probiotic supplementation, of which administration begins when chicks are placed on farms (Liao et al., 2012; Fathi et al., 2017; Gao et al., 2017).

Recently, the emergence of *in ovo* techniques made it possible to manipulate the intestinal bacteria colonization before chicks have even been hatched or exposed to farm environments (Pedroso et al., 2016; Roto et al., 2016; Teague et al., 2017; Hou and Tako, 2018). Our lab has previously used the *in ovo* technique as an experimental model to address how the early intestinal colonization shapes the microbiome composition in hatching chicks (Wilson et al., 2019). In that study, it was shown that different isolates provided *in ovo* resulted in different microbiome profiles at the day of hatch (DOH), suggesting that neonatal exposure to beneficial bacteria may be critical for influencing GIT populations throughout the life of poultry. Understanding how the introduction of pioneer species in the gut, regardless of long-term colonization, can lead to the modification of the microbiota toward beneficial bacterial growth has the potential to provide essential information about the development of the immune system, control of opportunistic bacteria and optimization of the functional properties of growth (Pedroso et al., 2016).

Therefore, it was hypothesized that manipulating the early colonization in the intestine might drive shifts in the composition and structure of the post-hatch microbiome, shaping microbial colonization patterns up to the mature microbiota. To test this hypothesis, we performed an *in ovo* application of two non-pathogenic *Enterobacteriaceae* isolates and LAB-probiotic to determine how the initial intestinal colonization can affect the development and establishment of the mature ileal microbiota.

## Materials and Methods

### Study Design

The trial was performed on fertile eggs from commercial Ross 708 broiler breeder flocks obtained from a local hatchery. As standard operating procedures, the eggs were sanitized before storage and incubation. All hatching eggs were incubated under standard conditions at the Ohio Agricultural Research and Development Center’s poultry research farm. Once eggs were confirmed fertile, at embryonic day 18, the air-cell end of each egg was treated with iodine (Povidone-Iodine 10% topical solution, Drug Mart, Medina, OH, United States) before a small hole was aseptically punched into the shell with an inoculation needle *in ovo* inoculations contained one of the following: 0.2 mL of 0.9% sterile saline (S), which served as the control group, or approximately 10^2^ cells of *Citrobacter freundii* (CF), *Citrobacter* spp. (C2) or LAB mixture (L) administered into the amnion. After inoculation, the eggs were allocated by treatments into three separate benchtop hatchers (Hova-Bator model 1602N, Savannah, GA, United States) per treatment, for a total of 12 hatchers, which had been disinfected with 10% bleach prior to use. Strains CF and C2 were selected from our previous study as non-pathogenic bacteria from the gut of healthy birds (Bielke et al., 2003), and the homology of strains was confirmed by next-generation sequencing (NGS). The L culture was composed of a mixed inoculum of *Lactobacillus salivarius* and *Pediococcus* ssp. Bacterial inoculum was prepared as described by Wilson et al. (2019). All experimental procedures were approved by The Ohio State University’s Institutional Animal Care and Use Committee (IACUC).

### Sample Collection

Immediately post-hatch, chicks were co-mingled on a treatment basis, and 128 chicks were placed into treatment-separated brooder battery cages with *ad libitum* access to a standard corn-soy diet and water (Nutrient Requirements of Poultry, 1994). At 3 days post-hatch, 20 chicks per treatment were randomly selected for the collection of ileal samples. Similarly, by 10 days of age, the remaining 12 chicks per treatment were removed. Since there were three mortalities in CF, only nine birds were sampled for this treatment. Chicks were euthanized via cervical dislocation, and samples were aseptically collected *post mortem*. Mucosal scrapings and digesta were collected from two different sampling sites in the ileum: (1) the region proximal of Meckel’s diverticulum, denominated as upper ileum (UI) and (2) the region proximal to the ileocecal junction and distal to the Meckel’s diverticulum, designated as lower ileum (LI). Ileal contents were placed into 1.5 mL tubes, flash-frozen in liquid nitrogen at the time of collection, and stored at −80°C until further use.

### DNA Extraction, Sequencing, and Data Processing

DNA was extracted from UI and LI samples following methods described by Yu and Morrison (2004). Briefly, digesta taken from each 3-day-old bird was weighed, in an equal amount of 0.125 g, then, was mixed to create pooled samples from two birds (*n* = 10). Ileal samples, collected at 10 days of age, were weighed in an equal volume of 0.25 g of digesta and placed directly into a single 2 mL tube (*n* = 12). Extraction of DNA was performed following the protocol of the Qiagen stool kit (Qiagen, Valencia, CA, United States) as recommended by the manufacturer with modifications described previously by Wilson et al. (2019). Once DNA was precipitated, it was stored in deionized water and measured spectrophotometrically by Synergy HTX multi-mode plate reader (BioTek U.S., Winooski, VT, United States).

The generation of the PCR amplicon was achieved by amplification of the V4–V5 regions of the 16S rRNA gene using 515F and 806R primers (515F: GTGYCAGCMGCCGCGGTAA, 806R: GGACTACHVGGGTWTCTAAT). DNA samples were library prepared for NGS using the Illumina MiSeq platform (2 × 300 bp; Illumina, San Diego, CA, United States) by The Ohio State University Molecular and Cellular Imaging Center (OSU MCIC).

A sequence quality screen was performed to ensure high-quality sequences submitted to the analysis pipeline. Briefly stated, sequence quality was determined using the FASTQC and MultiQC toolkits. Sequence reads exhibiting a quality score of lower than 20 were removed. Further, low complexity reads, those shorter than 200 bp in length, and mismatched primers were removed. Additionally, reads exhibiting low sequence qualities on either end were trimmed. The pre-processed FASTQ files were then imported to the QIIME2 platform for analysis. The main analytical steps were as follows: firstly, reads were de-multiplexed and classified into their respective samples. Next, additional sequence quality control measures and feature table construction were performed by the DADA2 algorithm implementation in QIIME2. Quality control measures eliminated reads with barcode errors, reads with more than two nucleotides mismatches, and chimeras. Sampling depth was set to 800 sequences per sample. The high-quality sequences emanating from the afore-mentioned quality control measures were subsequently clustered together using the q2-feature-classifier plugin with the GreenGenes 13.8 reference database. The resulting feature table was used to calculate diversity metrics and create abundance infographics.

Alpha-diversity was measured by Shannon’s diversity index, evenness, and richness. Richness was expressed as a number of observed OTUs. Beta-diversity was determined by applying unweighted and weighted UniFrac distance metric. Principal coordinate analysis (PCoA) was plotted to visualize similarities or dissimilarities along with the two sampling sites, age and between treatments. Relative abundance from the family to the genus-species levels was calculated for each *in ovo* treatment.

### Statistical Analysis

Kruskal–Wallis test was assessed to compare the differences in the microbial Shannon’s diversity index (H) across treatments and over time. To examine ileal microbiome beta-diversity variation among treatments at different ages and ileum sections, the permutational multivariate analysis of variance was conducted using distance matrices (PERMANOVA; QIIME2). All *P*-values were adjusted with the Benjamini–Hochberg procedure, up to 1,000 permutations, and had a false discovery rate less than 0.05. The mean relative abundances of microbial communities, richness, and evenness were analyzed by ANOVA and compared with a Student’s *t*-test (*P* < 0.05; JMP 12.2.0, SAS Institute Inc.). Additionally, the Pearson correlation coefficient (r) was applied to identify correlations between bacterial colonization patterns and age (R software version 3.4.1).

## Results

A total of 32,122,605 16S rRNA sequence reads was obtained. The number of mapped sequence reads of overall samples ranged from 842 to 384,833 with a mean of 111,536.82 (median 115,607).

Interestingly, inoculated strains did not remain in the GIT long-term. *L. salivarius* was significantly higher by 3 days in L compared to other treatments in UI. Similarly, it occurred in the LI, *L. salivarius* showed a meaningful reduction (*P* < 0.05) relative to S, CF, and C2 treatments by 10 days of age (Figure 7B and Supplementary Table 1). *Pediococcus* was rarely detected in the ileum during any of the sampling days. Correspondingly, in both ileal sites, *Citrobacter* had the highest population in CF treatment at 3 days of age (Figures 6A, 7A and Supplementary Table 1; *P* < 0.05). Whereas, it remained lowly detectable in C2. By 10 days of age, *Citrobacter* was not found in CF or C2 treatments.

### Alpha and Beta-Diversity in the Ileal Microbiome

To investigate whether different initial colonizers in the intestine could affect the ileal microbiome diversity, alpha-diversity was quantified by richness, evenness, and Shannon index, which relates both OTU richness and evenness by the total number of observed species. Biodiversity of microbial communities was defined at the two ileal sampling sites, where the overall alpha-diversity of samples within UI was similar to LI (*P* = 0.101; Supplementary Figure 1).

**FIGURE 1:**
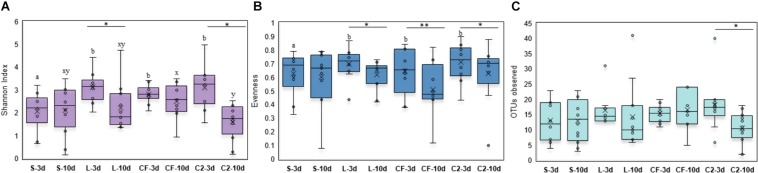
Alpha-diversity indices measured by **(A)** Shannon index, **(B)** Evenness, and **(C)** OTUs observed in upper ileum samples treated with either saline (S), LAB-probiotic (L), *Citrobacter freundii* (CF) or *Citrobacter* spp. (C2). Superscripts at the top of each index represent significant differences between treatments at 3 (a,b) and 10 days of age (x,y). Comparisons over time of collection within a treatment were reported with (^∗^) indicating significance at level *P* < 0.05 and (^∗∗^) a trend of significance (*P* = 0.056).

**FIGURE 2:**
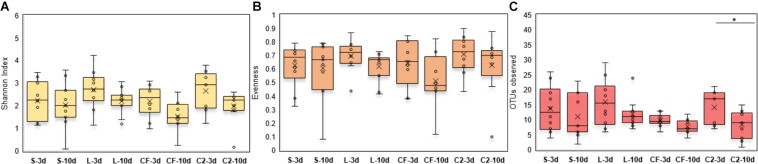
**(A)** Diversity, **(B)** Evenness, and **(C)** Richness in lower ileum microbiota treated with either saline (S), LAB-probiotic (L), *Citrobacter freundii* (CF) or *Citrobacter* spp. (C2). Comparisons over time of collection within a treatment were reported with (^∗^) indicating significance at the level of *P* < 0.05.

Within-sample microbial diversity among treatments in each ileal site was examined. In UI, Shannon diversity was statistically increased in L, CF, and C2 compared to S treatment (Figure 1A) at 3 days of age. Consistent with Shannon index, evenness was decreased in S, in which over 88% of individuals belonged to only two genera, reducing the number of species present at this age (Figure 1B). As shown in Figures 2A–C, the alpha-diversity measurements in LI were statistically similar among all groups by 3 and 10 days of age.

Next, diversity shifts in each *in ovo* treatment over time were compared. Interestingly, L and C2 treatments significantly decreased in diversity and evenness by 10 days only in UI (Figures 1A,B). In terms of richness, based on the observed OTUs, C2 treatment was lower by 10 days in UI (*P* < 0.05; Figure 1C) and LI (Figure 2C). These results indicate that LAB and the *Enterobacteriaceae* strains as pioneer colonizers in the ileum may be an important driver of shifts in the microbial communities’ diversity.

**FIGURE 3:**
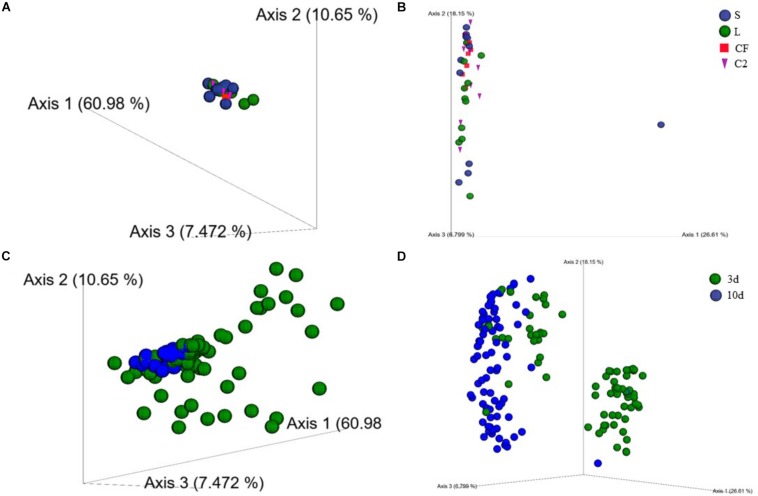
**(A)** Beta-diversity of microbial communities in lower ileum of broilers treated with either saline (S), LAB-probiotic (L), *Citrobacter freundii* (CF) or *Citrobacter* spp. (C2) at 10 days of age based on weighted UniFrac distances. **(B)** Principal coordinate analyses (PCoA) plot is showing the beta-diversity clustering pattern of samples in lower ileum at 10 days of age derived from unweighted UniFrac (*P* < 0.05, PERMANOVA). **(C)** Weighted and **(D)** Unweighted UniFrac-based PCoA plots reveal a clustering by age in upper and lower ileum (*P* < 0.05, PERMANOVA).

To characterize the phylogenetic composition of bacterial communities among samples, the UniFrac distance matrix was analyzed. Distance-based PERMANOVA analyses (Weighted UniFrac distances) in LI showed that there was a significant separation of the gut microbiome of broilers exposed to LAB-probiotic *in ovo* from those of CF, C2, and S treatments (Figure 3A; C2 × L *P*-value = 0.002; CF × L *P*-value = 0.004; S × L *P*-value = 0.001). At the same age, the LI bacterial communities in L, S, and CF treatments showed a dissimilarity between samples according to unweighted UniFrac distance analyses (*P* < 0.05; PERMANOVA), although it was not evident in PCoA plots (Figure 3B). Based on these findings, it can be assumed that exposure to either LAB-probiotic or *Enterobacteriaceae* was able to modify the structure of microbial communities up to mature microbiota.

Additionally, results in the principal coordinate plots of weighted (Figure 3C) and unweighted UniFrac (Figure 3D) showed heterogeneity among samples from 3 and 10 days of age, demonstrating an expected clustering by age (*P* < 0.05). The beta-diversity metrics also detected a larger variation in the community structure along with the two sampling sites in the ileum. Weighted pairwise PERMANOVA revealed that bacterial communities from L and CF were phylogenetically unrelated to each other among the two ileum sections at 3 and 10 days of age (*P* < 0.05; Supplementary Figure 2). Therefore, the early exposure to different bacterial isolates may influence the degree of similarity between microbial communities across the two different sites of the ileum.

### Taxon Abundance

In order to assess whether LAB-probiotic or *Enterobacteriaceae* acting as pioneer colonizers could have an impact on specific ileal bacteria populations, the analysis was completed via a taxonomic approach to follow the bacterial load dynamics at family and genus-species levels across 3 and 10 days of age. Among observed OTUs in the entire dataset, unidentified bacteria represented 10.23 and 2.65% of the total sequences obtained in UI and LI, respectively, at 3 days of age (data not shown). Conversely, by 10 days of age, most of the sequences were taxonomically identified from the kingdom down to the genus. When we examined the OTUs that were generated at this age, less than 0.5% of relative bacterial abundance was not classified beyond the domain.

**FIGURE 4:**
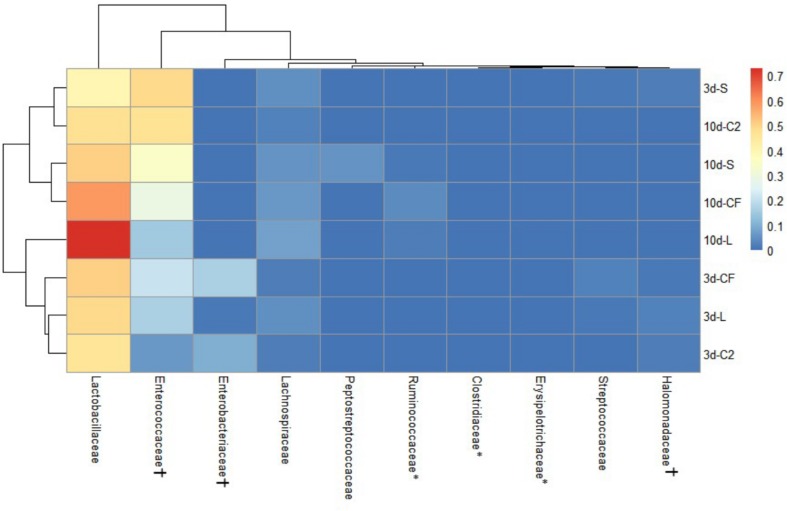
Double hierarchical dendrogram of bacterial distribution within *in ovo* treatments over time in upper ileum. The taxonomic association of bacterial families is depicted in the columns. Hierarchical clustering in the rows is based on the composition similarity between treated samples. Heatmap plot represents the mean relative percentage of each bacterial family within samples treated with either saline (S), LAB-probiotic (L), *Citrobacter freundii* (CF) or *Citrobacter* spp. (C2) at 3 or 10 days of age. Statistical differences (*P* < 0.05) between groups were reported for each bacterial population (†) by 3 days of age and (^∗^) 10 days of age.

**FIGURE 5:**
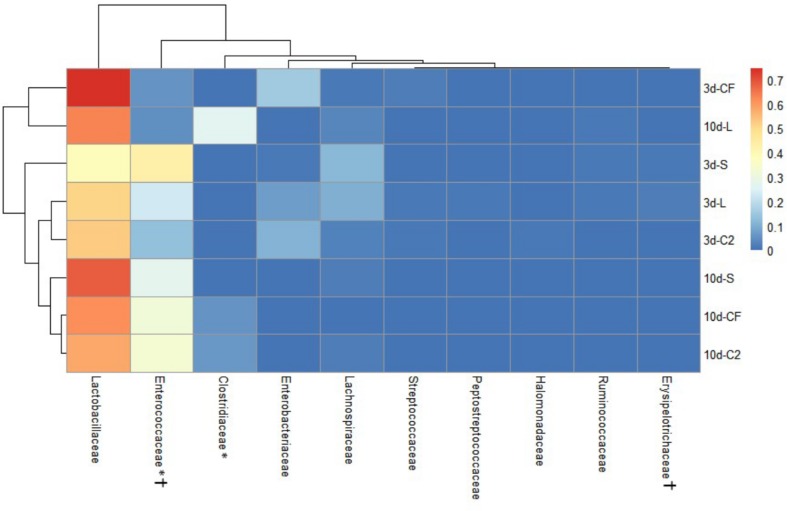
Dual hierarchical dendrogram based upon the predominant bacterial families identified in lower ileum microbiota of broilers treated with either saline (S), LAB-probiotic (L), *Citrobacter freundii* (CF) or *Citrobacter* spp. (C2). The taxonomic association of bacterial families is represented in the columns. Hierarchical clustering in the rows is based on composition similarity between treated samples. Heatmap plot represents the mean relative percentage of each bacterial family within samples. Statistical differences (*P* < 0.05) between groups were reported for each bacterial population (†) by 3 days of age and (^∗^) 10 days of age.

The ileal microbiota by 3 and 10 days of age appeared to be dominated by *Lactobacillaceae* without significant differences among groups (Figures 4, 5). Interestingly, the microbiota population of *Enterococcaceae* was highly variable based on bacterial isolate *in ovo* treatment. By 3 days of age, *Enterococcaceae* was significantly reduced in L (16.23%), CF (20.71%), and C2 (6.18%) relative to S (48.92%) in UI (Figure 4). Likewise, it occurred in LI, which *Enterococcaceae* population was statistically lower in CF (5.49%) and C2 (13.10%) compared to S (43.19%) and L (22.98%) by 3 days of age. While by 10 days of age, only in L treatment had *Enterococcaceae* abundance markedly decreased to 4.87% (*P* < 0.05; Figure 5). Similarly, hierarchical cluster analysis indicated that L microbial composition by 10 days of age had a clear separation from CF, C2, and S, suggesting a unique shaping of the bacterial community structure in birds exposed to LAB-probiotic *in ovo* (Figure 5).

In addition, our study has shown that the *Enterobacteriaceae* abundance varied greatly, resulting in substantial population differences among the pioneer colonizers groups at 3 days of age (Figures 4, 5). In our prior report, *Enterobacteriaceae*, as well as *Citrobacter* showed to be dominant in CF and C2 at DOH (Wilson et al., 2019). Over time though, this study shows that the populations became less abundant in UI (CF: 16.11 and C2: 9.73%; Figure 4) and LI (CF: 15.49 and C2: 11.06%; Figure 5). By 10 days of age, OTUs belonging to *Enterobacteriaceae*, found in CF and C2 decreased to 0.01% and became statistically similar among treatments in UI and LI. This finding means that even though *Enterobacteriaceae* was one of the pioneer colonizers in these treatments, it was transient, and as the bird developed, the population decreased to low levels, in some cases below detectable limits in the ileal microbiota.

**FIGURE 6:**
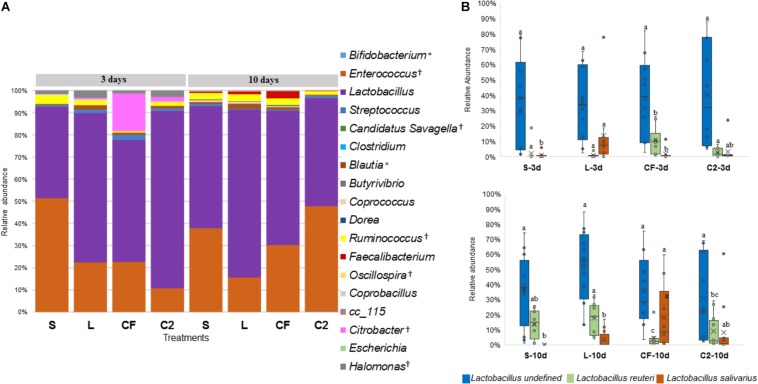
**(A)** Genus-level taxonomic distribution among samples in upper ileum. Bars represent the mean relative percentage of each bacterial genus within samples treated with either saline (S), LAB-probiotic (L), *Citrobacter freundii* (CF) or *Citrobacter* spp. (C2). Statistical differences (*P* < 0.05) between groups were reported for each specific bacterial population (†) by 3 days of age and (^∗^) 10 days of age. **(B)** The box plot shows the *Lactobacillus* species relative abundance distribution within *in ovo* treatments over time in upper ileum. Statistical differences (*P* < 0.05) for each species population between groups were reported with different letters.

**FIGURE 7:**
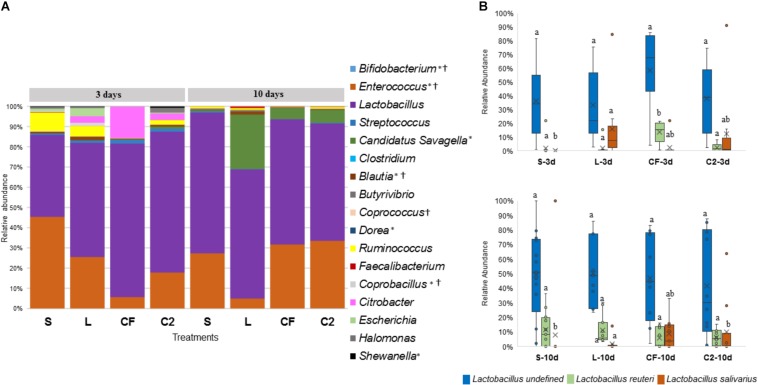
**(A)** Microbiome composition by genus-level in lower ileum microbiota of broilers treated with either saline (S), LAB-probiotic (L), *Citrobacter freundii* (CF) or *Citrobacter* spp. (C2). Bars represent the mean relative percentage of each bacterial genus within samples. Statistical differences (*P* < 0.05) between groups were reported for each specific bacterial population (†) by 3 days of age and (^∗^) 10 days of age. **(B)** The box plot represents the relative of abundance *Lactobacillus* species within *in ovo* treatments over time in lower ileum. Statistical differences (*P* < 0.05) for each species population between groups were reported with different letters.

The abundance of *Lactobacillus*, a widespread member of poultry ileal microbiota, was not affected by time, sampling site, or treatment (*P* > 0.05; Figures 6A, 7A). Undefined *Lactobacillus* was the most prevalent across the treatments, however, significant differences were identified only for *Lactobacillus reuteri* and *L. salivarius* abundances (Figures 6B, 7B).

In relation to *Blautia*, a butyrate producing bacteria, there was an evident increase seen in L (1.42%) by 3 days in LI samples (S: 0.43%, CF: 0.81%, C2: 0.58%, *P* < 0.05; Figure 7A). Similarly, at 10 days of age, L had a higher population of *Blautia* relative to S, CF, and C2 (*P* < 0.05) in UI (S: 0.85, L: 2.07%, CF: 1.03%, C2: 0.52%; Figure 6A) and LI (S: 0.54%, L: 1.68%, CF: 0.15%, C2: 0.42%; Figure 7A). Additionally, by 10 days, the LI samples showed a higher presence of *Dorea* in L (0.30%; *P* < 0.05) than any other *in ovo* treatment (S: 0.16%, CF: 0.06%, C2: 0.09%; Figure 7A).

*Candidatus Savagella* was rarely detected in UI, with a fraction of only 0–0.15% (Figure 6A). By 3 days of age in LI, OTUs from *Candidatus Savagella* were statistically similar between treatments, in which populations reached a downward of 1%. However, by 10 days, the taxonomic abundance shifted. The LAB-probiotic treatment showed the highest prevalence of *Candidatus Savagella* (26.57%) relative to S, CF, and C2 (0.39, 5.51, and 6.26%, *P* > 0.05, respectively; Figure 7A). There was a consistent distribution of OTUs belonging to *Candidatus Savagella* across all samples in L treatment.

### Correlation Between Microbial Composition and Age of Broilers

**FIGURE 8:**
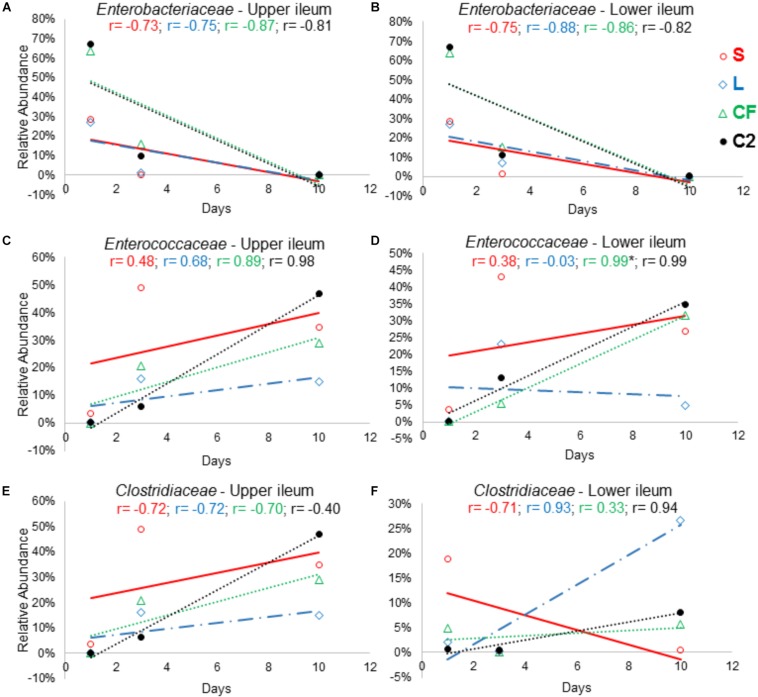
The relationship between broiler age and ileal microbial colonization pattern. Panels **(A)**, **(B)**, **(C)**, **(D)**, **(E)**, and **(F)** show the Pearson correlation levels in specific ileum site between *Enterobacteriaceae*, *Enterococcaceae*, and *Clostridiaceae* among all *in ovo* treatments (represented by colors) and aging (hatch, 3 and 10 days of age). Correlation with significance (*P* < 0.05) is represented by (^∗^).

In order to further assess the effect of different pioneer colonizers on age-related changes in ileal microbiota composition, the top six family level OTUs detected in the four treatments were identified (Supplementary Table 2). Bacterial relative abundance data from DOH, previously published by Wilson et al. (2019) was added into the analysis, and a Pearson correlation analysis to determine the influence of early colonization of *Enterobacteriaceae* or LAB on the microbial colonization dynamics was completed. Values of r revealed that, regardless of *in ovo* pioneer colonizer exposure, *Enterobacteriaceae* was negatively correlated with time in both ileum habitats (Figures 8A,B). The negative relationship indicates that the reduction of *Enterobacteriaceae* in ileum is linked to the age of bird and occurs prior to the maturation of microbiota.

On the other hand, an increase in relative abundance of *Lactobacillaceae* in the ileum was shown to be age-dependent. Among all treatments, a positive relationship was observed between the *Lactobacillaceae* population and bird age. In LI, this relationship was statistically significant (*r* = 0.998; *P* = 0.041) in samples from L treatment (Supplementary Table 2).

The results demonstrated that *Clostridiaceae* colonization pattern hardly changed among groups as birds aged. In LI, *Clostridiaceae* relative abundance was positively associated with time in L, CF and C2 (*r* = 0.936, *r* = 0.334, *r* = 0.946; *P* > 0.05). In contrast, in UI, all relationships between *Clostridiaceae* relative abundance and age were negative (Figure 8E).

The majority of the relationships between the *Enterococcaceae* population and aging were positive (Figures 8C,D). Although, in the L this relationship was negative, differing from those that were inoculated with saline or *Enterobacteriaceae* in LI (*r* = −0.03, *P* = 0.982; Figure 8D). Therefore, this finding suggests that LAB as pioneer colonizer may have a major influence on *Enterococcaceae* colonization during the establishment of mature microbiota.

## Discussion

This study examined the impact of early intestinal colonization by different bacterial species on the development and persistence of ileal microbiota. An important outstanding question was whether and how these microbes, as first intestinal bacterial settlers, could shape microbiome abundances and diversity over time. Importantly, the findings presented here demonstrate that the pioneer intestinal colonization plays a critical role in driving the course of diversity and composition of microbial communities in the ileum of 10-day-old broilers.

One of the most interesting findings was the decrease in the alpha-diversity measurements in the ileum when examined over time. The UI microbiome of L and C2 was less diverse from three through 10 days of age. Indeed, it is expected that maturation of intestinal microbiota keeps diversity relatively stable over time (Donaldson et al., 2017; Reese and Dunn, 2018). Preliminary data from our laboratory has identified a distinct diversity pattern in the ceca, where all *in ovo* treatments displayed an increase in biodiversity through time, as described by Wilson et al. (2019). Nevertheless, limitations such as using pooled samples at 3 days and single samples from 10-day-old chicks may have led to a degree of variation in our analysis. To our knowledge, the drop in ileal diversity was potentially accounted for by the relative overabundance of few dominating species as bird aged. Another candidate mechanism that may influence intestinal evenness is the presence of highly competitive microbial communities (Bauer et al., 2018). Exactly how probiotic strains can interact in the intestinal ecosystem, synergistically inhibiting, or promoting the growth of selected microbial populations (Aminnezhad et al., 2015). Our results demonstrated that the increase of *Lactobacillaceae* abundance in ileum was shown to be age-dependent. By 10 days of age, *Lactobacillaceae* also dominated the microbial communities in the LI among the different treatments; however, only in L treated birds, there was an overabundance of another functionally relevant microbial member. Additionally, weighted UniFrac distance and hierarchical cluster analysis showed a dissimilarity between L samples from those found in CF, C2, and S, which reflected in a singular microbial community in L. Collectively, it was presumed that LAB-probiotic as pioneer colonizers might shape the early microbial communities by selecting for persistent heterogeneous functioning symbionts up to mature microbiota.

In face of declining biodiversity, the host-microbiota may lose redundant species (Foster et al., 2017), which means that members of the community who are phylogenetically unrelated, but have similar functional niches can be substituted for one another with little impact on the ecosystem (Lozupone et al., 2012; Tian et al., 2017). Strong evidence suggests that the inoculation of a LAB-probiotic resulted in changes in *Enterococcaceae* colonization pattern as the birds aged. We cogitate that LAB-probiotic may have influenced a reduction of functional redundancy of lactic acid producers, given that when the *Enterococcus* population decreased, the *Lactobacillus* compensated for the loss by increasing in abundance (notably in the UI). This issue highlights the complexity of microbe-microbe interactions in the intestinal ecosystem, and it might be worthwhile to explore further with metabolic and host function approaches to elucidate this assumption.

Approaching the colonization patterns over time allowed for the identification of succession and persistence of critical microbial communities as *Enterobacteriaceae* in the different ileal sites. The predominance and endurance of most species of *Enterobacteriaceae* within the intestine is a standard marker of dysbiosis (Rivera-Chávez et al., 2017), even though it is well-known that this family is a pioneer colonizer in the gut (Donaldson et al., 2017). In addition, members of *Enterobacteriaceae* may also delay or block the growth of beneficial microbiota due to their utilization of important early ecological niches (Pedroso et al., 2016). In previous work, *Enterobacteriaceae* species were dominant in the CF microbiome at hatch (Wilson et al., 2019) and potentially related to inducing the intestinal inflammation signaling pathway found in this treatment. However, by 10 days of age, when the stability of microbial communities’ dynamics is expected, *Enterobacteriaceae* abundance was identified to be negatively correlated with aging and was absent or decreased to 0.1% among all treatments. These results strongly suggest that *Enterobacteriaceae* persistence in the ileum was not influenced by the bacterial isolates that embryos were exposed *in ovo*, although its high relative abundance at an early age affected the microbiome composition at 3 days of age, postponing the mature microbiota establishment. In agreement of this study, Juricova et al. (2013) demonstrated that chicks infected with *Salmonella* Enteritidis at one and 4 days of age delayed the microbiota development because the number of *Enterobacteriaceae* population increased, while *Clostridiales* and *Lactobacillales* relative abundances decreased.

The decline of the *Enterobacteriaceae* population can be linked to the succession of anaerobic microorganisms along with intestinal maturation, and fermentative metabolism carried out in the ileum (Matamoros et al., 2013). Components of normal microbiota, as *Lactobacillaceae* a highly prevalent member of ileal microbiota during the poultry lifetime, produce short-chain fatty acids and/or lactic acid (Rivière et al., 2016), which contribute to the inhibition of many acid-sensitive bacteria, such as *Enterobacteriaceae*, by lowering the pH of the intestinal contents (Cisek and Binek, 2014). These results were in agreement with Donaldson et al. (2017) and Ballou et al. (2016), who observed that *Enterobacteriaceae* colonization in chickens did not last until the late microbiota.

Metagenomics analysis identified an apparent stimulation of segmented filamentous bacteria (SFB) abundance in ileal microbiota of LAB-probiotic treated broilers. The primary site for SFB in chickens, a *Clostridiaceae* member, designated recently as *Candidatus Savagella* (Thompson et al., 2013), is in the mucosa of LI (Liao et al., 2012). SFB has been allied with inducing maturation of all immune system components. On one side, SFB may mediate stimulation of Th17 cells and T-cell responses, boost pro-inflammatory cytokine and immunoglobulin A production, but on the other side, immune-enhancing SFB may trigger inflammation disorders (Ivanov et al., 2009; Ivanov and Littman, 2010; Chung et al., 2012; Buffie and Pamer, 2013). Besides, SFB has been positively related to body weight gain in commercial turkeys (Danzeisen et al., 2013) and chickens (Johnson et al., 2018).

Segmented filamentous bacteria colonization is time-dependent in broilers, presumably because they are related to immune system maturation, which occurs with the aging of the host (Liao et al., 2012). This statement is consistent with our results, which found a positive correlation among *Clostridiaceae* colonization patterns and time-course up to 10 days of age in L treatment (Figure 8F). In previous work, it was demonstrated that broilers treated with probiotic seemed to have an anticipation of SFB intestinal colonization (Liao et al., 2012). However, supplementation of *Lactobacillus* strains as an approach to shaping the host immune response by enhancing SFB abundance was not effective in mammals (Fuentes et al., 2008). Chung et al. (2012) reported that SFB alone was not capable of causing any significant mucosal protection from bacterial invasion in the mouse intestinal microbiota, indicating that SFB might be coevolved with another symbiont to support adaptive immunity in the mammalian intestine.

In light of our results, we suggest that the establishment and enhancement of SFB may have been promoted by a synergistic effect of early exposure of LAB strains. This work supports the postulation that selected pioneer colonizers in the intestine may determine the microbial colonization course (de Oliveira et al., 2014; Pedroso et al., 2016; Roto et al., 2016). Additionally, to the best of our knowledge, no studies have indicated that LAB-probiotic *in ovo* inoculation promoted an enhancement of SFB in the intestinal microbiota.

## Conclusion

In conclusion, these results can provide a more comprehensive insight into the role of intestinal pioneer colonizers in driving the course of microbial community composition and diversity over time. Our analysis demonstrated that the early bacterial colonization did not necessarily become colonized resident gut microbiota, but they did influence the microbiome development. *Enterobacteriaceae* strains, as pioneer colonizers, delayed the microbial consortium establishment in the ileum, whereas, LAB supported selection alongside greater colonization of ileal symbiotic populations of young broilers. Finally, an essential contribution of this study is that *in ovo* LAB-probiotic inoculation serves as a potential study model for SFB functions within poultry microbiota ecology and host-associated immune responses.

## Data Availability Statement

The sequencing datasets for this study are available at Sequence Read Archive under BioProject accession number PRJNA552855.

## Ethics Statement

The animal study was reviewed and approved by The Ohio State University’s Institutional Animal Care and Use Committee (IACUC).

## Author Contributions

KW, WB, AD, and KC carried out the project. DR, EW, and KW performed the analyses. DR interpreted the results and wrote the manuscript in consultation with LB. All authors contributed to experimental design, discussed the results, and commented on the manuscript.

## Conflict of Interest

The authors declare that the research was conducted in the absence of any commercial or financial relationships that could be construed as a potential conflict of interest.
