# Supplementary material for: Intestinal Pioneer Colonizers as Drivers of Ileal Microbial Composition and Diversity of Broiler Chickens
Source: Front Microbiol. 2020 Jan 9;10:2858. doi: 10.3389/fmicb.2019.02858 (PMC6962117; doi:10.3389/fmicb.2019.02858)
Supplement: Supplementary file 1 [file Data_Sheet_1.docx]

*Supplementary Material*


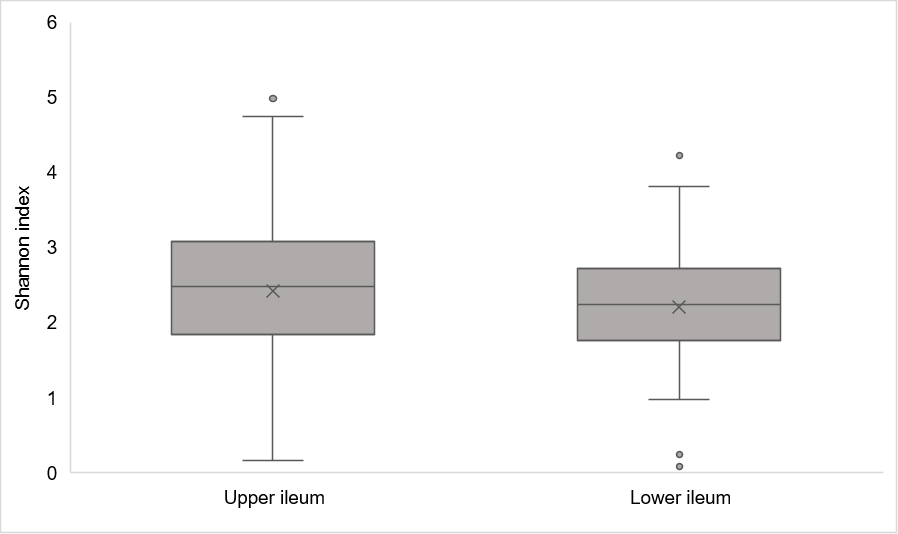


**Supplementary Figure 1.** Alpha-diversity in upper and lower ileum samples


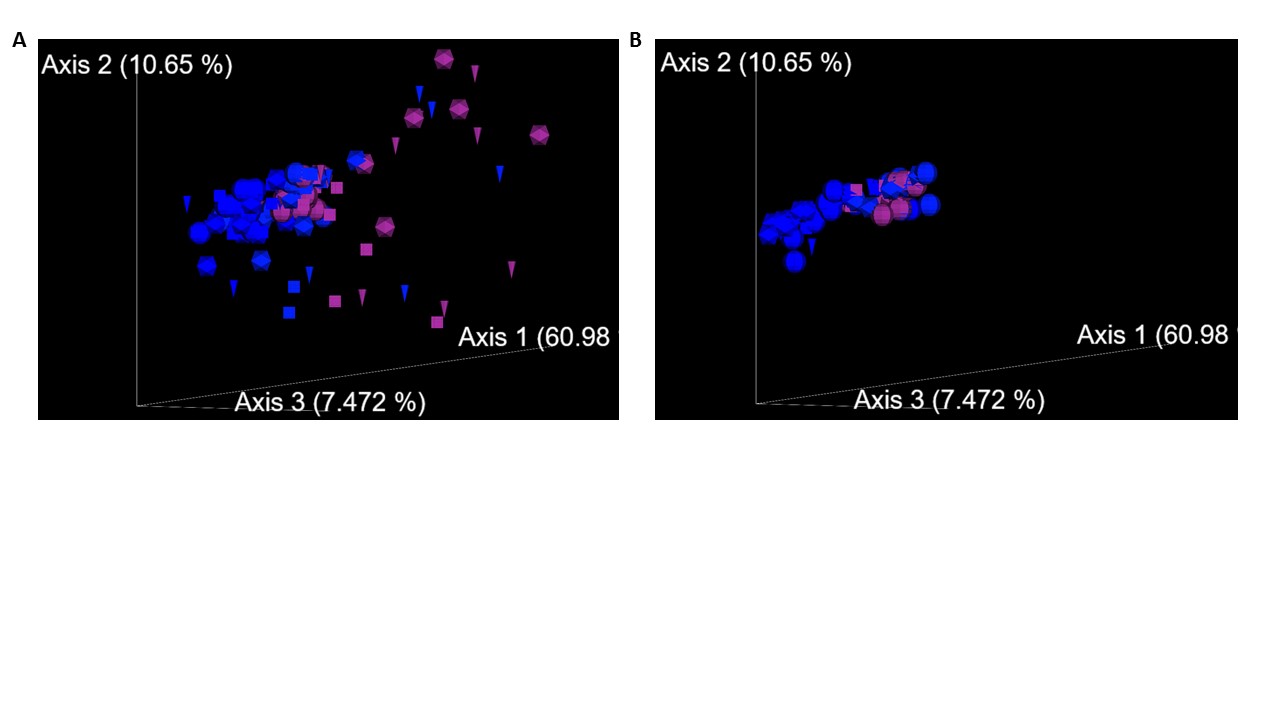


**Supplementary Figure 2**. Beta-diversity along the two sampling sites upper (purple) and lower (blue) ileum of broilers treated with either saline (S; sphere), LAB-probiotic (L; icosahedron), *Citrobacter freundii* (CF; cube) or *Citrobacter* spp. (C2; cone) at 3 (**A**) and 10 (**B**) days of age derived from weighted UniFrac.
